# Facilitation or Competition? Tree Effects on Grass Biomass across a Precipitation Gradient

**DOI:** 10.1371/journal.pone.0057025

**Published:** 2013-02-22

**Authors:** Aristides Moustakas, William E. Kunin, Tom C. Cameron, Mahesh Sankaran

**Affiliations:** 1 Institute of Integrative and Comparative Biology, Faculty of Biological Sciences, University of Leeds, Leeds, United Kingdom; 2 School of Biological and Chemical Sciences, Queen Mary, University of London, London, United Kingdom; 3 Department of Ecology and Environmental Science, Umeå University, Umeå, Sweden; 4 National Centre for Biological Sciences, TIFR, GKVK Campus, Bangalore, India; Helmholtz Centre for Environmental Research – UFZ, Germany

## Abstract

Savanna ecosystems are dominated by two distinct plant life forms, grasses and trees, but the interactions between them are poorly understood. Here, we quantified the effects of isolated savanna trees on grass biomass as a function of distance from the base of the tree and tree height, across a precipitation gradient in the Kruger National Park, South Africa. Our results suggest that mean annual precipitation (MAP) mediates the nature of tree-grass interactions in these ecosystems, with the impact of trees on grass biomass shifting qualitatively between 550 and 737 mm MAP. Tree effects on grass biomass were facilitative in drier sites (MAP≤550 mm), with higher grass biomass observed beneath tree canopies than outside. In contrast, at the wettest site (MAP = 737 mm), grass biomass did not differ significantly beneath and outside tree canopies. Within this overall precipitation-driven pattern, tree height had positive effect on sub-canopy grass biomass at some sites, but these effects were weak and not consistent across the rainfall gradient. For a more synthetic understanding of tree-grass interactions in savannas, future studies should focus on isolating the different mechanisms by which trees influence grass biomass, both positively and negatively, and elucidate how their relative strengths change over broad environmental gradients.

## Introduction

Savannas are ecosystems characterised by a continuous grass layer and a discontinuous tree layer. They cover over 20% of the Earth's total land surface and support a significant proportion of the planet's livestock and wild herbivores [1], [2]. The ratio of trees to grasses in savannas can vary depending on several environmental factors with precipitation and soil properties generally considered the predominant drivers at large scales [1], [2], [3], [4], [5] which in turn modulate plant-plant interactions at local scales [6], [7], [8].

Traditionally, ecologists have emphasized the role of competition between trees and grasses as being a key determinant of savanna structure [1], [9], [10]. While the importance of competition in structuring ecological communities is widely recognized, there is also a growing appreciation of the role of facilitation amongst plants in structuring ecological communities [11], [12], [13], [14], especially in stressful environments [15], [16]; indeed facilitation is a process that needs to be more integrated into ecological theory [17]. Facilitation can occur through various mechanisms including refuge from physical stress [18], refuge from predation [17], refuge from competition [19], and improved resource availability [20].

At present, the importance of tree-grass facilitation relative to competition, and the role of microhabitats and microclimatic factors created by the presence or absence of trees for grasses in savannas has not yet been fully explored (but see [7], [21], [22], [23], [24], [25]). While it is well established that trees in savannas compete with grasses for light, nutrients, and water [6], [26], there are also several cases where trees have been reported to have facilitative effects on grasses [21], [23], [27], [28]. For example, grass biomass has been found to be higher under tree canopies when compared to the interspaces between trees in several systems [21], [28], [29], [30], [31], [32]. However, grass biomass has also been reported to be lower beneath tree canopies in some savannas [31], [33], [34], while other studies have found no differences in grass biomass beneath tree canopies and in tree interspaces [35]. At present, the reasons underlying these divergent responses are unclear.

Savanna trees can facilitate grasses by altering resource availability and microclimatic conditions, and providing grasses refuges from grazing. Trees in particular affect water redistribution in the landscape [24], and play important roles by creating shade [21], [23], [36] and by drawing water from deep sources inaccessible to grasses, i.e. hydraulic lift [21], [23], [35]. However, the extent to which such positive effects offset the negative effects of competition is unclear. For example, in a study of hydraulic lift (the process of water movement from relatively wet to dry soil layers through plant roots) of *Acacia tortilis* it was found that the δ^18^O of water extracted from the xylem water of grasses indicated that when they grew near trees they had values similar to those of groundwater either because grasses could use water from deeper soils or because they used water hydraulically lifted by trees [37]. However, at the same site [35] found lower soil moisture content under trees than in the open, both during dry and wet seasons, and marginally higher grass biomass in open areas. Thus, while hydraulic lift did facilitate water uptake by grasses, the effects of competition with tree roots cancelled the beneficial influence of tree roots on grass biomass at that site [38]. Overall, findings in savannas have shown that both facilitation and competition can occur in the same ecosystem, and that competitive and facilitative effects do not always balance [39].

In this study, we examined the effects of isolated trees on grass biomass across a precipitation gradient in an African savanna. Specifically, we looked at how grass biomass changed as a function of distance from the base of the tree and with tree size, and how this relationship was influenced by precipitation. In arid and semi-arid savannas where water is the main limiting resource [2], [40], we expected trees to facilitate grasses by enhancing water availability, and predicted that grass biomass values would be higher in the sub-canopy areas than in tree interspaces. In contrast, in more mesic savannas, where water is typically less limiting and factors such as shading by trees becomes increasingly important, we expected grass biomass to be lower in sub-canopy areas than in the interspaces between trees. Further, since the microclimate experienced by grasses is also likely to be affected by individual tree characteristics such as size, we expected that the extent to which trees facilitate or compete with grasses would change with tree size. Increases in tree size can lead to increased soil resource availability and hence increased sub-canopy grass biomass as a result of hydraulic lift or increased nutrient contents below canopies [41]. Alternately, increases in tree size can also result in increased solar radiation and evapotranspiration in sub-canopy areas leading to lowered soil moisture and sub-canopy grass biomass [21], [23]. We expected sub-canopy grass biomass to increase with tree size with such effects particularly pronounced in mesic areas where sub-canopy grasses tend to be light-limited, and where increased solar irradiation beneath larger trees can in fact have a positive effect on sub-canopy grass biomass.

## Methods

### Study area

The study was conducted in the Kruger National Park (KNP), South Africa between January and February 2008. The park is situated in the savannas of north-eastern South Africa, and covers an area of ∼19,633 km^2^. Altitude ranges from 260 to 839 m above sea level within the park. Mean Annual Precipitation (MAP) varies from around 750 mm in the south to approximately 350 mm in the north, with marked annual variations [42]. The vegetation in the park is characterized by dense savanna with species such as *Acacia nigrescens*, *Sclerocarya birrea*, *Combretum imberbe*, *Colophospermum mopane*, *Terminalia sericea* and *Burkea africana* dominating the canopy depending on the location within the park [42].

Our study was conducted in the long-term Experimental Burn Plots (EBPs) of the Kruger National Park [42]. Only ‘unburnt’ treatment plots were sampled (i.e. fire exclusion plots) for this study. Plots were located in four major landscapes of the park underlain by both granites: Pretoriuskop (MAP = 737 mm) and Skukuza (MAP = 550 mm), and basalts: Satara (MAP = 544 mm) and Mopane (MAP = 496 mm). In each of the four landscapes, there were four replicate plots, each covering an area of ∼7ha. Fire had been excluded from our study plots for more than 50 years [42], [43]. All necessary permits for field work were obtained from the Administration, Scientific Services and the local Rangers of the Kruger National Park.

### Sampling methodology

We identified isolated trees in each of the replicate plots (N = 16; four in each landscape). Isolated trees were defined as those for which distance to the nearest woody plant (tree or shrub) neighbour was as least three times the canopy radius of the focal tree. For each tree, we recorded height, girth at breast height, and canopy diameter along two perpendicular axes. In cases where measuring girth at breast height was not feasible we recorded their girth at the closest available point. In addition, we also measured grass biomass at different distances from the base of the tree, corresponding to 50%, 100% (i.e. canopy edge or drip line), 150% and 200% of the tree canopy radius [30]. Distances were measured as a proportion of canopy size rather than as absolute values in order to facilitate comparison of canopy effects on grass biomass of uneven-sized trees [30]. For each tree, grass biomass was measured along 3 transects radiating away from the base of tree, each 120^0^ apart from the other. Grass biomass measurements were taken at peak herbaceous standing crop in January and February. Biomass values from the three transects were averaged to get a mean value for grass biomass for each distance category for each tree. Grass biomass was estimated using a disc pasture meter [44] specifically calibrated for KNP [42], [45]. Disc pasture meter calibrations, conducted using samples from across the full extent of KNP, indicate a high degree of concordance between measured and estimated values of grass biomass in KNP (R^2^ = 0.972, 45). Grass biomass was estimated from disk pasture meter readings (in cm) using the formula y = −301.9+226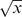, where x is the disc reading in cm and y the grass biomass in g/m^2^ [45]. For a more detailed description of the device and its calibrations for KNP see [44], [45].

In all, a total of 93 trees were sampled across all plots (Table 1). One plot (Numbi block in Pretoriuskop) had no isolated trees as per our criterion as a result of the dense nature of the vegetation in the plot.

**Table 1:** Details of the study sites.

Site	MAP	N	Height [StDev]	Dominant isolated tree species
Mopane	496	17	4.29 [1.53]	*Colophospermum mopane* (15)
Satara	544	28	5.87 [1.85]	*Acacia nigrescens* (19)
				*Acacia burkei* (6)
Skukuza	550	27	6.17 [1.65]	*Sclerocarya birrea* (10)
				*Combretum apiculatum* (4)
				*Terminalia sericea* (4)
				*Combretum collinum* (3)
Pretoriuskop	737	21	5.02 [1.30]	*Terminalia sericea* (20)

MAP is the mean annual precipitation of the site in mm. N is the number of isolated trees sampled at each site. Each site is replicated by four blocks. The mean height of sampled trees [Height] and standard deviation [StDev] in meters is also listed, as is the identity, and number sampled (in parentheses), of the dominant tree species at each site. The data gathered here have also been included in a larger dataset comprising similar data from Africa and North America as part of a larger-scale analysis of competitive-facilitative interactions in savannas [31].

### Statistical Analysis

Linear mixed effects models were used to quantify the effects of tree characteristics on grass biomass across sites. Rainfall, distance from the base of the tree, and tree size were the factors included in the analysis (fixed effects), with geology (granite or basalt) included as a random effect in the model. We chose to include geology as a random rather than fixed effect because the design of the Experimental Burn Plots in KNP does not allow separating out the individual and interactive effects of both rainfall and geology simultaneously; the two lower rainfall sites (Mopane and Satara) are on basalts and the two higher rainfall sites (Skukuza and Pretoriuskop) are on granites. Our dataset also contained a different number of species at each site. Because sample sizes were limited, we were not able to explicitly test to see if tree effects on grass biomass varied depending on tree species identity. However, since tree species identity can potentially play a role in regulating tree-grass interactions in savannas [3], [6], [46] we additionally included tree species identity as a random effect in our analysis. An initial calculation of the contribution of the random structure (Standard Deviations of the random effects from our model) showed that the factor that contributes least to variance in the estimates of the mean grass biomass is geology (Random effects: *∼1|geology*, Intercept StdDev = 17.73082; *∼1 | geology/plot*, Intercept StdDev = 114.0258; *∼1 | geology/plot/species*, Intercept StdDev = 106.3738; *∼1 | geology/plot/species/treeID*, Intercept StdDev = 54.58133). A separate evaluation of the contribution of species identity using a linear NULL model, (*ANOVA(biomass∼plot/species/treeID*) where the nested structure is a fixed effect, showed that there is a greater contribution to the variance in grass biomass from plots within sites (σ^2^ = 14646) or between individual trees within species (σ^2^ = 24232) than between tree species (σ^2^ = 3023).

In the mixed effects model used, grass biomass data were grouped by distance increments within individual trees nested by species within plots, within geology to account for non-independence of data from trees on the same site [47]. Data grouping accounts for autocorrelation between samples in all its forms [47]. Thus, although we only report on the effects of trees on grass biomass independent of tree species identity, our analysis nevertheless accounted for the fact that there are differences in tree species composition across our study sites.

We evaluated the effects of three correlated measures of tree size – height, basal area and canopy area – on grass biomass across sites. We created three different maximal models on non-transformed grass biomass with different combinations of the correlated fixed effects: tree height, canopy area and basal area (as indices of tree size) as well as distance from the base of the tree and site. The maximal models included all possible interaction terms. We used the Akaike Information Criterion (AIC) to assess the best maximal model. Of the three indices of tree size evaluated, the maximal model which included tree height as the index of tree size was the best, and we only report results from this model here. The maximal model which included tree height as the index of tree size was subsequently simplified via stepwise deletion wherein non-significant factors and their interactions were sequentially removed, until further simplification was not justified. Model selection was conducted using the AIC with maximum likelihood estimation, but the presented fit of the minimal model used restricted maximum likelihood (REML); [47]. Any deletion that did not increase AIC scores by more than 2 was deemed to be justified [48]. The minimum adequate model selected by AIC or comparative F-tests were identical. Inspection of residual plots for constancy of variance and heteroscedasticity indicated that the model was well behaved in all cases. All analyses were conducted in R 2.14.1 using the nlme package [49].

## Results

The minimum adequate model explaining grass biomass included the main effects of rainfall, distance and tree height and the two-way interactions between rainfall and distance, and rainfall and height (Table 2).

**Table 2:** ANOVA results of the most parsimonious linear fixed effects model.

Variable	numDF	denDF	F-value	p-value
(Intercept)	1	270	98.99	**<.0001**
Rainfall	3	54	14.31	**<.0001**
Distance	3	270	37.49	**<.0001**
Height	1	54	0.41	0.5241
Rainfall∶Distance	9	270	8.51	**<.0001**
Rainfall∶Height	3	54	2.72	0.0532*

**Figure 1:**
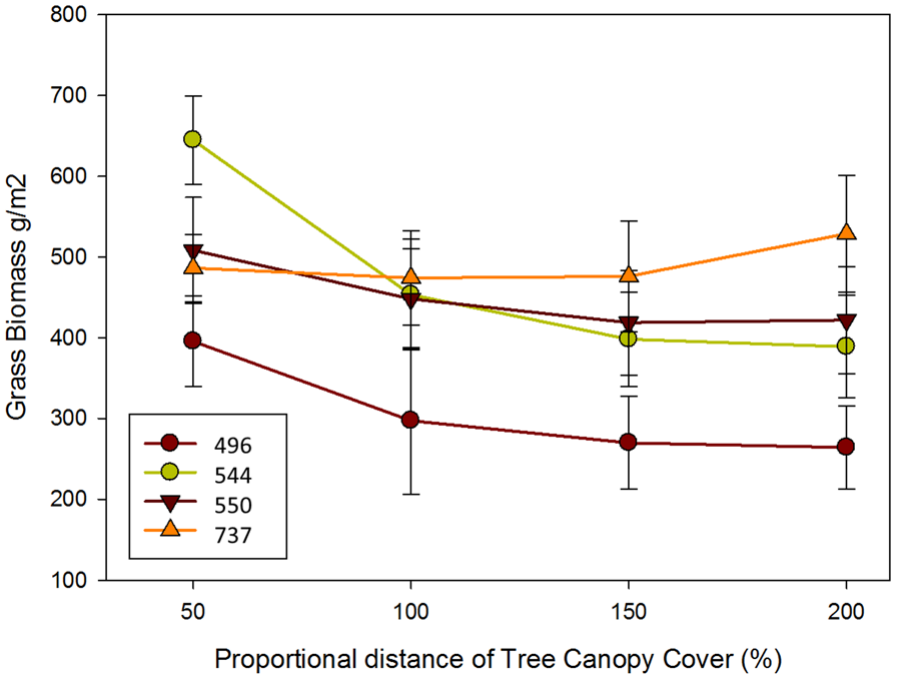
Grass biomass in each of the four MAP values replicated (corresponding to the four different study sites each replicated by four blocks) as a function of distance from the base of the tree (stem) corresponding to 50%, 100%, 150% and 200% of the tree canopy radius. Biomass values at 100% correspond to the canopy edge or drip line. Bars represent ±2 standard errors. Grass biomass is greater beneath the canopy (50% distance) compared to outside the canopy at the three drier sites, but not at the wettest site.

**Figure 2:**
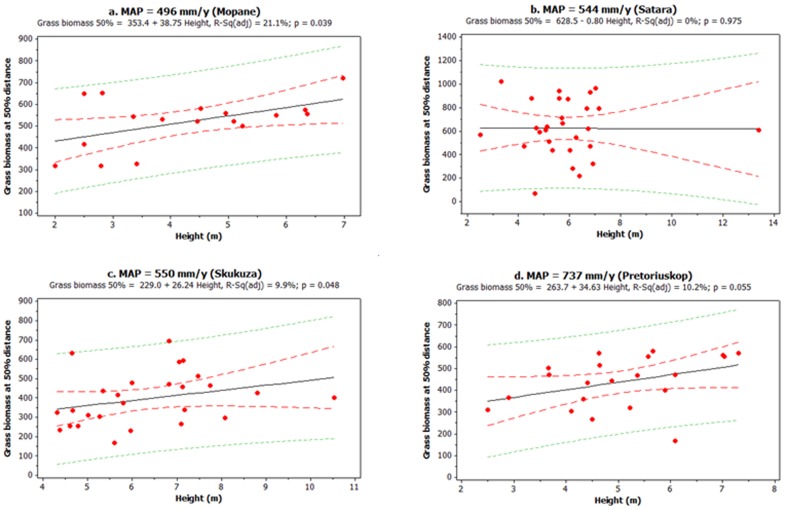
Sub-canopy grass biomass (g.m^−2^) as a function of tree height (m) at each of the four study sites. Sites are ordered in terms of increasing rainfall from Mopane (a) to Pretoriuskop (d). Sub-canopy grass biomass refers to grass biomass measured at 50% of the canopy radius, i.e. half way between the base of the tree and canopy edge. Solid lines are the best fit regression, dashed lines the 95% confidence interval, and the dotted lines the 95% predicted interval. Removal of the tallest tree in Satara as an outlier does not qualitatively change the outcome of the regression.

Grass biomass is the dependent variable while fixed effects are distance from the base of the tree, tree height, and rainfall. The model includes random effects of individual trees, nested in tree species identity, nested within strings nested in geology (see statistical analysis). Significant p-values are bolded. The three-way interaction between Rainfall∶Distance∶Height as well as the two-way interaction between Distance∶Height were included in the maximal model but were simplified as non significant. According to our results, tree height is not a significant contributor *per se*, but the interaction between Rainfall∶Site is marginally significant. Thus, height is a potentially significant contributor depending on rainfall. Given the fact that Table 2 shows 2 two-way interactions as significant, (Rainfall∶Distance and Rainfall∶Height) we need two figures to assess this. Figure 1 shows the relationship between Rainfall∶Distance and Figure 2 between Rainfall∶Height.

Tree effects on grass biomass beneath canopies changed across the rainfall gradient. In the three drier sites (Mopane MAP = 494 mm, Satara MAP = 544 mm, & Skukuza MAP = 550 mm), grass biomass was significantly higher beneath tree canopies than outside canopies (Table 2 and Figure 1). In contrast, there were no significant differences in grass biomass beneath and outside canopies at the wettest site (Pretoriuskop, MAP = 737, Fig. 1).

**Figure 3:**
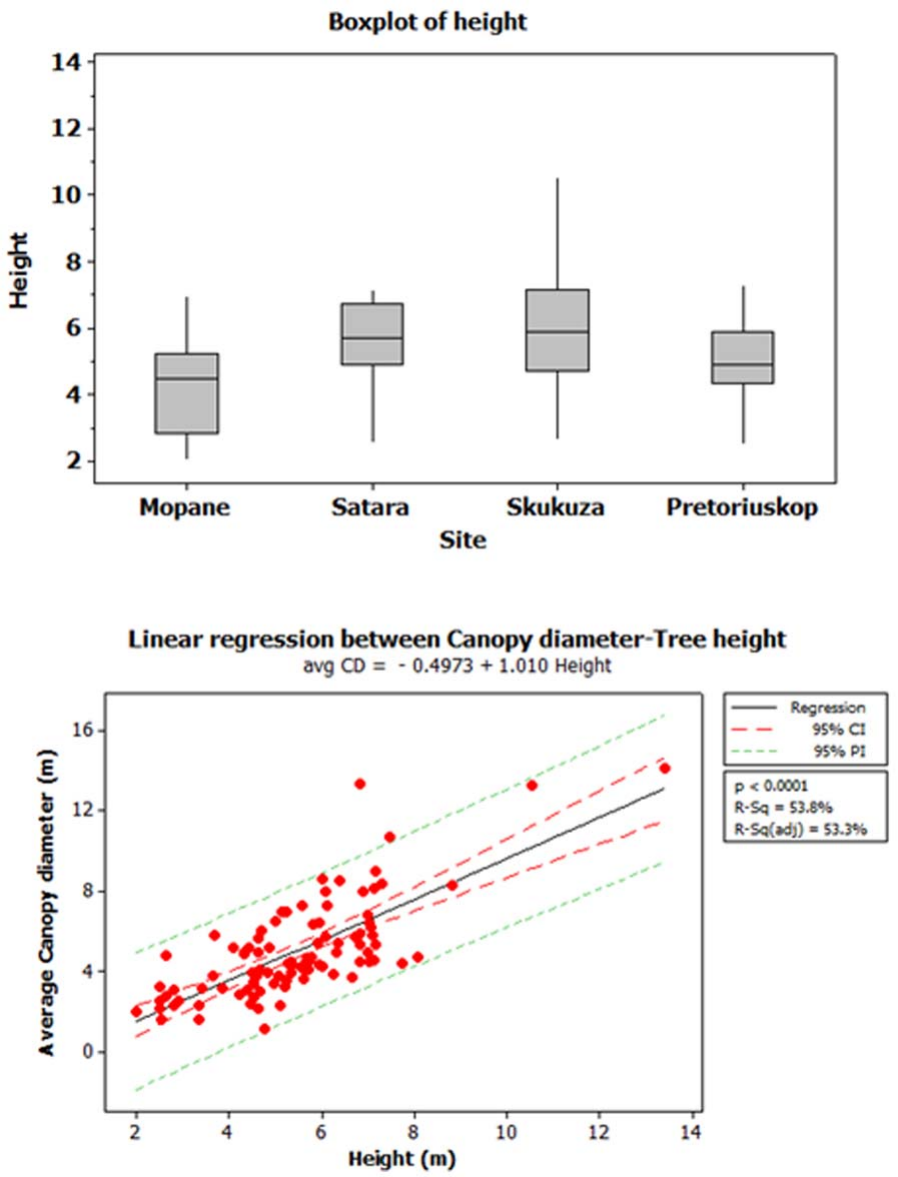
Box and whisker box plots of tree heights in the different study sites (a). The solid line is the median, and the boxes are defined by the upper and lower quartile (25^th^ and 75^th^ percentiles). The whiskers extend up to 1.5 times the inter-quartile range of the data. The figure indicates that distribution of tree heights was not uneven between study sites. Relationship between average canopy diameter (m) and tree height (m) across all sampled trees (**b**). Average canopy diameter is the mean of canopy diameters measured along two perpendicular axes. Regression results indicate a tight relationship between canopy diameter and tree height, with taller trees having proportionally larger canopies regardless of species identities (adjusted R^2^ = 53.8%, p<0.0001. CI: 95% confidence interval, PI: 95% predicted interval).

The interaction between rainfall and tree height was marginally significant (P = 0.053) suggesting that effects of tree height on grass biomass differed between sites (rainfall x height interaction, Table 2). At the driest site (Mopane), sub-canopy grass biomass increased as tree height increased (adjusted R^2^ = 21.1%, p = 0.039, Figure 2a). A similar pattern was observed at Skukuza (adjusted R^2^ = 9.9%, p = 0.048; Figure 2c). At the wettest site (Pretoriuskop) grass biomass below tree canopies increased with tree height but that relationship was only marginally significant (adjusted R^2^ = 10.2%, p = 0.055; Figure 2d). At Satara there was no effect of tree height on grass biomass (adjusted R^2^ = 0%, p = 0.975; Figure 2b). These observed patterns were not a consequence of consistent differences in tree architecture or height resulting from species turnover across sites; tree height distributions were similar across our four study sites (Figure 3a), with taller trees having proportionally larger canopies regardless of species identity (Figure 3b).

## Discussion

Our results indicate that the nature of tree-grass interactions changes from positive to negative across a gradient of increasing precipitation. We suggest that this change occurs due to a decline in the relative importance of facilitation of grasses by trees, relative to competition between them, with increasing rainfall.

According to our results, the net impact of trees on grass biomass appears to shift qualitatively between 550 (Skukuza) and 737 (Pretoriuskop) mm MAP (Table 1 and Figure 1) in our study site. This is in accordance with the results of previous studies showing that tree effects on grass biomass are more positive on arid sites than in mesic ones [23], [27]. Grass biomass has been reported to be higher below tree canopies in more arid savannas (MAP<∼650 mm; e.g. [21], [28], [30]), and lower below tree canopies in more mesic sites (MAP>∼650 mm; e.g. [34], [50]). In sites with intermediate rainfall (MAP≈650 mm), grass biomass did not appear to be significantly different beneath and away from tree canopies e.g. [35], [37].

Our results are also in accordance with previous empirical and theoretical studies of facilitative-competitive interactions from other systems which suggest that the relative importance of facilitation versus competition should vary across gradients of abiotic stress, with facilitation encountered in more stressful environments. [51], [52]. Facilitation, or positive interactions across plant communities, has been reported across precipitation [51], [52] altitudinal [53], [54], temperature [53], and slope [7] gradients. Although we have not specifically quantified the mechanisms underlying the observed patterns in our study, we suspect that it is predominantly a result of improved water conditions beneath trees in arid sites. Other studies have argued that facilitative and competitive effects are density-dependent rather than driven by only abiotic factors; low plant densities are favourable for facilitative effects, while increased plant densities favour competition [39]. Of course, the latter is often correlated with abiotic factors, and higher plant densities are usually encountered in areas of lower abiotic stress. In a meta-analysis of plant interactions in arid environments it was found that the effects of neighbours was density-driven with positive net effects of neighbours occurring at low abiotic stress and negative effects at high stress [15]. Likewise, a study conducted at an area of MAP = 500 mm reported that few, isolated trees had positive local effects on savanna grasses, but in areas of high tree density the negative landscape-scale effects of trees outweighed these positive effects [55]. Our results show that switches from facilitation to neutrality or competition can also occur independent of density dependence since our results are based exclusively on isolated trees. A potential explanation for the absence of facilitation in the more mesic sites is that tree Leaf Area Index (LAI) tends to increase with increasing rainfall [56], [57], and thus mesic sites are likely to be associated with lower light penetration through tree canopies, with potential negative impacts for shade intolerant C_4_ grasses.

Within the overall precipitation-driven pattern, our results indicate that understorey grass biomass can be additionally influenced by tree characteristics such as height. For example, tree height was a significant factor influencing grass biomass in our most arid study site, explaining up to 21% of the variance in sub-canopy grass biomass. To the best of our knowledge, very few studies have examined the effect of tree height on tree grass interactions, and those that have report contrasting patterns. In a study examining the relationship between tree height and grass biomass in savannas, no relationship was found between grass biomass, tree height, and distance from the canopy, despite soil nutrient concentrations being much higher under larger trees [58], [59], on the other hand, examined the effects of *Colophospermum mopane* trees on understorey vegetation, and found grass biomass to be higher below tree-canopies, with effects more pronounced under large canopies. Similarly, in a study where tree age was included as a factor potentially mediating tree effects on grass biomass, older trees were found to facilitate grasses more than younger trees, and this was attributed to the fact that older trees had a higher fraction of deep rather than lateral roots [7]. In contrast, [33] found that taller *Acacia karroo* trees suppressed grasses more than shorter ones in a semi-arid savanna in South Africa.

Tree size can influence sub-canopy grass biomass by i) altering soil resource availability in sub-canopy areas, ii) modulating solar irradiation and hence evapotranspiration and soil temperatures in sub-canopy areas, and/or iii) regulating access to grazers and thus influencing grass offtake from sub-canopy areas [21], [27], [58], [60]. Increases in tree size can lead to increased soil resource availability and hence increased sub-canopy grass biomass as a result of hydraulic lift or increased nutrient contents below canopies [41], and such effects may be manifest in both arid and mesic sites. However, increases in tree height can also result in a higher canopy and thus increased solar radiation and evapotranspiration in sub-canopy areas leading to lowered soil moisture and sub-canopy grass biomass, particularly in arid areas where water is the limiting resource. Similarly, in mesic areas where sub-canopy grasses are predominantly light-limited, increased solar irradiation beneath larger trees can in fact have a positive effect on sub-canopy grass biomass. Finally, increased grazing pressure as a result of greater access to sub-canopy grasses beneath taller trees can result in sub-canopy grass biomass decreasing with tree size [60], [61], [62], with such effects more pronounced in arid and semi-arid areas which typically support higher grazer biomass [63]. Ultimately, the net effect of increasing tree size on sub-canopy grass biomass is contingent on the relative strengths of these different processes. The lack of a consistent relationship between tree size and sub-canopy grass biomass in our study suggests that the relative strengths of these different processes varied differentially across the rainfall gradient in our study area.

The design of the long-term experimental burn treatments at KNP, where the drier sites occur on basalts and the wetter sites on granites, did not allow us to explicitly isolate the effects of geology from the larger scale rainfall driven patterns. However, our results indicate that the contribution to the overall variance accounted for by including geology as a random effect was relatively small when compared to that resulting from differences between individual trees within a species, differences between tree species and between replicate plots within a site. Furthermore, the overall pattern of greater grass biomass beneath tree canopies compared to tree interspaces was evident at both Satara and Skukuza, sites which are characterized by similar rainfall but differ in their underlying geologies. Future studies that explicitly sample across comparable, broad rainfall gradients on both granites and basalts, will help determine the extent to which the rainfall driven switch from facilitation to competition is influenced by underlying geology.

Our results indicate that the net effect of savanna trees on grasses is contingent on environmental context, with facilitation outweighing competition in arid sites and competition predominating in more mesic sites. Although our analysis did not examine how the nature of tree-grass interactions changes over time, interactions in plant communities can also switch between positive and negative contingent on temporally-generated gradients at the same location too; for example as a result of seasonal changes in climate, grazing pressure, etc. [64], [65]. Future studies should focus not only on isolating the different mechanisms by which increases in tree size influence grass biomass and production and how this changes across broad environmental gradients, but also on the extent to which such effects change over time and are dependent on tree species identity. This will provide for a more integrated understanding of tree-grass interactions in savannas.
